# Hypoxia, Metabolism and Immune Cell Function

**DOI:** 10.3390/biomedicines6020056

**Published:** 2018-05-15

**Authors:** Ewelina Krzywinska, Christian Stockmann

**Affiliations:** 1 Institut National de la Santé et de la Recherche Médicale (INSERM), Paris Cardiovascular Research Center, Unit 970, 56 Rue Leblanc, 75015 Paris, France; 2 Institute of Anatomy, University of Zurich, Winterthurerstrasse 190, CH-8057 Zurich, Switzerland

**Keywords:** oxygen, hypoxia, HIF signaling pathway, oxygen metabolism, immune cells, innate immune responses, adoptive immune responses, immunity, inflammation

## Abstract

Hypoxia is a hallmark of inflamed, infected or damaged tissue, and the adaptation to inadequate tissue oxygenation is regulated by hypoxia-inducible factors (HIFs). HIFs are key mediators of the cellular response to hypoxia, but they are also associated with pathological stress such as inflammation, bacteriological infection or cancer. In addition, HIFs are central regulators of many innate and adaptive immunological functions, including migration, antigen presentation, production of cytokines and antimicrobial peptides, phagocytosis as well as cellular metabolic reprogramming. A characteristic feature of immune cells is their ability to infiltrate and operate in tissues with low level of nutrients and oxygen. The objective of this article is to discuss the role of HIFs in the function of innate and adaptive immune cells in hypoxia, with a focus on how hypoxia modulates immunometabolism.

## 1. Hypoxia-Induced Transcriptional Machinery

The oxygen concentration is closely associated with cellular proliferation, division and survival, and is generally maintained by homeostatic mechanisms operating at the cellular and systemic organ (tissue) levels [1,2,3,4,5,6]. The hypoxic response in cells and tissues is coordinated by a family of hypoxia-inducible transcription factors HIFs (HIF-1, HIF-2 and HIF-3 [1,7,8,9,10,11,12]. HIF expression can be generated by hypoxia itself, but also by others circumstances associated with pathological stress including cancer, inflammation, and bacteriological infection. HIF-1 is expressed and is detected in immune cell populations including macrophages, neutrophils, dendritic cells, as well in T and B lymphocytes and immune lymphoid cells (ILC1, ILC2 and ILC3). HIF-2 expression is also detected in a range of cell types, including endothelial cells and certain immune cells including myeloid cells, as well as lymphoid cells in response to hypoxia and cytokines [3,13].

### HIFs

**Figure 1:**
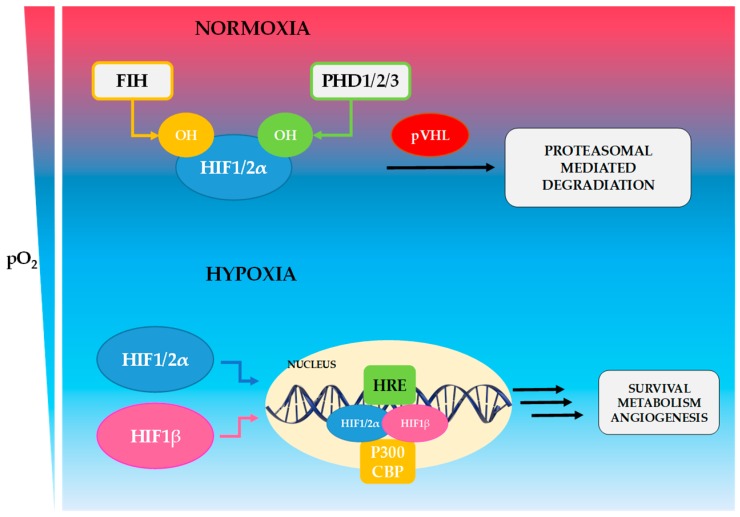
Regulation of HIF pathway. Under normoxia, PHDs (PHD1, PHD2 and PHD3) and factor inhibiting hypoxia-inducible factor (FIH) hydroxylate the HIF-1α and HIF-2α. This hydroxylation facilitates HIFα binding to the von Hippel-Lindau (VHL) E3 ubiquitin ligase complex, leading to fast ubiquitination and proteasomal degradation. During hypoxia, PHDs and FIH are inhibited by the absence of oxygen, in consequences, hypoxia reduces HIFα hydroxylation and leading to HIFα stabilization and activation. Once stabilized, HIFα subunit is translocated to the nucleus, where formed a complex with HIF-1β, then recruit coactivator p300/CBP, and upon binding to the consensus hypoxia response elements (HRE) within target genes, involved in a large type of processes, as cellular metabolism, proliferation, differentiation, cell survival, migration, apoptosis or angiogenesis.

HIFs are a family of dimeric transcription factors composed of an oxygen-sensitive α-subunit (HIF-1α, HIF-2α or HIF-3α) and of a constitutively expressed β-subunit (HIF-1β, HIF-2β, and HIF-3β). α- and β-subunits consist of basic helix-loop-helix (bHLH) and PER-ARNT-SIM homology (PAS) domains that mediate dimerization and form transcriptional active complex. HIF-β subunits are members of the arylhydrocarbon receptor nuclear translocator (ARNT) family, they are also known as ARNT1, ARNT2, or ARNT3, respectively [7,14]. HIF-1α is the best described and the most often expressed isoform, whereas HIF-2α is expressed predominantly in the heart, lung, kidney and placenta [15]. Little is known about HIF-3α, which is expressed mainly by epithelial cells in the lung and the kidney [16]. HIF acts as a transcriptional regulator of genes with promoters and enhancers that contain hypoxia response element sequences (HREs) [7,17]. HIF-α stability is regulated posttranscriptionally by oxygen-dependent prolyl hydroxylase domain enzymes (PHD1, PHD2 and PHD3) as well as by asparaginyl hydroxylase enzyme FIH (Factor Inhibiting HIF). PHDs and FIH are O_2_ sensors and their enzymatic activity is proportional to changes in pO_2_ levels. This system of HIF regulation provides rigorous control of the hypoxic response over a wide range of pO_2_ [18]. Under normoxia, when oxygen is available, PHDs hydroxylate two specific proline residues (Pro402, Pro564) in the O_2_-dependent degradation domain (ODD), which leads to decreased transcriptional activity for HIF. This hydroxylation facilitates HIF-α binding to the von Hippel–Lindau (VHL) E3 ubiquitin ligase complex, leading to fast ubiquitination and proteasomal degradation of the α subunit (as shown in Figure 1) [3,8,19,20,21]. FIH hydroxylates a specific asparagine residue (Asn803) in the C-terminal transactivation domain (CTAD) of HIF-α subunit and decreases the transcriptional activity of HIFs. The Asn803 hydroxylation in the CTAD prevents p300 from binding, and prevents HIF target gene expression [18,22]. In addition to oxygen, PHDs and FIH use as co-substrate 2-oxoglutarate (2-OG, also called α-ketoglutarate) and as cofactors iron (Fe^2+^) and ascorbic acid (vitamin C) [8,23]. It was demonstrated that FIH has a lower Km for oxygen than the PHD enzymes and is still an active hydroxylase at lower oxygen levels.

During hypoxia, when the oxygen level drops, PHDs and FIH become inactive, resulting in HIF-α stabilization. Once stabilized, the HIFα subunit translocates to the nucleus, forms a complex with HIF-1β, then recruits the co-activator p300/CBP, and upon binding to the HRE within target genes that control a large number of processes, including cellular metabolism, proliferation, differentiation, cell survival, migration, apoptosis or angiogenesis (as shown in Figure 1) [3,8,23,24].

## 2. Hypoxia and Immunometabolism

HIFs are the most important regulators of metabolic adaptation to hypoxia and play a crucial role in modulating immune cell effector functions [25,26,27]. Therefore, we want to review how metabolic reprogramming by the HIF signaling pathway drives the function of different immune cell subsets. Immunometabolism is now considered as an indispensable regulator of immunity, and it seems to constitute another layer of immune cell control by HIFs.

Immune cells are resident or may be recruited from the oxygen-rich circulatory system to physiologically hypoxic (intestinal mucosa or lymphoid tissue) or pathologically hypoxic immune environment (tumors, infected, inflamed or ischemic tissues). Consequently, in physiologically hypoxic environments, HIFs contribute to innate and adaptive immune cell homeostasis, whereas in pathological hypoxia, HIF signaling can trigger tissue damage and immune cell dysfunction [2]. Hypoxia and the HIF signaling pathway impact on immune cell fate and function [28], including the up-regulation of glycolytic gene expression [29] after activation of different immune cells [25]. An adequate immune response requires rapid ATP production by using aerobic glycolysis [30] in order to generate precursors for the synthesis of lipids, amino acids and nucleotides that are necessary for rapid cellular proliferation and effector function [27,31]. Yet, it is likely that “immunometabolism” is more than just ATP production and it can be assumed that metabolic remodeling drives the phenotype of immune cells in many different ways [28,32]. Importantly, immune cell function can be altered via the impact of HIF on various other metabolic pathways than glycolysis, such as fatty acid synthesis, the tricarboxylic acid cycle, the pentose phosphate pathway, or amino acid metabolism [27].

**Figure 2:**
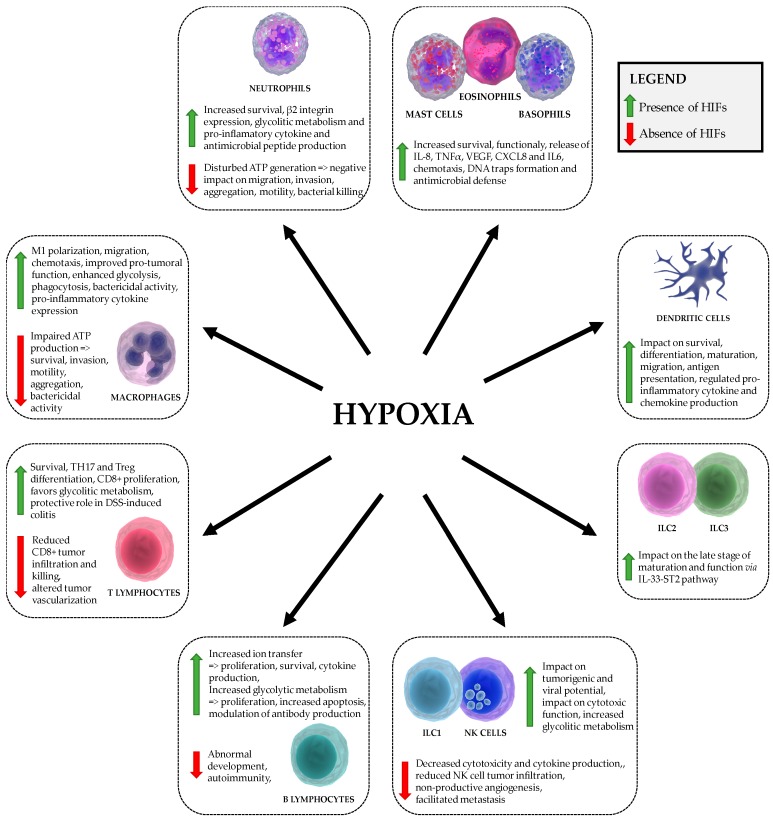
Regulation of innate and adaptive immunity by hypoxia and HIF signaling pathway.

Taken together, the HIF signaling pathway can modulate metabolism and function of various immune cell subsets (as shown in Table 1 and Figure 2) as described here in detail for each cell type.

## 3. The Effect of Hypoxia on Myeloid Cell Function and Metabolism

### 3.1. Hypoxia and Granulocytes

#### 3.1.1. Neutrophils

Neutrophils are short-lived granulocytes, which are the first leukocytes to migrate from the circulating blood to injured or infected sites. Their crucial role is to kill pathogens via the release of antimicrobial granules, activating cytokines like TNF-α, IL-1, interferons, defensins, and reactive oxygen species [33,34,35,36]. Moreover, neutrophils remove cellular debris [37] and they are able to generate the extracellular traps to degrade virulence factors and kill bacteria [38]. In addition to activating other immune cells, neutrophils can also dampen immune responses and promote the resolution of inflammation [39,40]. Similar to macrophages, neutrophils can be polarized into N1 or N2 subpopulations with differential abilities to cytokine production. It is known that, N1 neutrophils preferentially express IL-12 in response to lipopolysaccharide, whereas N2 neutrophils express IL-33 and IL-13 in response to helminth infections [41]. They also secrete IL-17 in response to fungal stimulation [36] or IFN-γ after bacterial infection [42]. Metabolic reprogramming in response to immune stimuli was first observed and described in neutrophils in 1959 by Sbarra and Karnovsky [43].

Several publications have documented that activation of the HIF signaling pathway in murine and human neutrophils increases their survival, β2 integrin expression, production of antimicrobial peptides and glycolytic metabolism [44,45,46,47,48]. Consequently, the absence of HIF-1α in neutrophils inhibits ATP generation and reduces their ability to invade tissues and kill bacteria [45,49,50]. Previous research has documented also that neutrophils play a very important role in resolution of intestinal inflammation [51,52,53], but the outcome of neutrophil-cell-specific deletion of HIF-1α in vivo during colitis is for the moment poorly understood. Thompson et al. [54] demonstrated that neutrophil-cell-specific deletion of HIF-2α increases their apoptosis in vivo, which affects neutrophilic inflammation and tissue injury in humans, mice, and zebrafish.

Neutrophils use high rates of Warburg-like glycolysis for the generation of ATP in which HIF-1α plays a crucial role in regulating the expression of key glycolytic enzymes [25,45]. In the absence of HIF-1, the intracellular ATP pools are reduced resulting in profound impairment of the inflammatory response due to decreased neutrophil aggregation, motility, bacterial killing in mice and humans [45,49]. Current research shows also that the stimulation of neutrophils with LPS augment the glucose uptake and oxygen consumption. This increased oxygen consumption is necessary for the production of H_2_O_2_ and pathogen clearance [112]. To summarize, all these results highlight the importance of a functional HIF signaling pathway in neutrophils for metabolic reprogramming and neutrophil function.

#### 3.1.2. Mast Cells, Basophils and Eosinophils

Mast cells, basophils and eosinophils were discovered by Paul Ehrlich at the end of the nineteenth century and they play a central role in allergic reactions and in the protection against parasite infection [113]. Mast cells produce a vast arsenal of biological substances (such as histamine, proteases, chemotactic factors, cytokines and metabolites of arachidonic acid), which act on the endothelium, smooth muscle cells, the connective tissue, mucous glands and other inflammatory cells in the airway. After multiple allergen challenge, the effects of these mediators are harmful to the host and contribute to several pathological aspects of airway inflammation and remodeling [114,115,116].

Basophils represent less than 1% of peripheral blood leukocytes and they show similarity to mast cells. Basophils play a very important role as central effector cells in TH2-mediated allergic responses, autoimmune disorders and microbial infections. They produce cytoplasmic granules, in which they store the effector molecules, including histamine, cysteinyl-leukotrienes and antimicrobial peptides. Upon activation, they are able to produce several chemokines as well as large amounts of IL-4, IL-6, IL13, which are TH2 associated cytokines [116,117,118].

Eosinophils are considered as important effector cells in several chronic allergic and hypersensitivity diseases. They play a very important role in host resistance to parasites (helminths), but they also possess antimicrobial activity. Upon activation, they release granules that contain the preformed enzymatic and nonenzymatic cationic proteins such as the major basic protein 1 and 2 (MBP1 and MBP2), eosinophil peroxidase (EPX), eosinophil cationic protein (ECP), and eosinophil-derived neurotoxin (EDN). Eosinophils have very important functions in airway inflammation and they contribute to tissue damage during asthma. Eosinophils are inducers of a chronic TH2 cell-mediated inflammation by the recruitment of TH2 cells through the release of CCL17 and CCL22 and also through eosinophil-DC interactions [116,119,120,121].

Current research shows that HIF-1α stabilization plays a crucial role for sustaining basophils, eosinophils and mast cell survival and function in mice and humans [58]. Moreover, the activation of the HIF signaling pathway is necessary in controlling the synthesis of IL-8 and TNF-α by mast cells following TLR ligand stimulation [55]. The activation of HIF-1α in mast cells has also a big impact on the expression of VEGF, CXCL8 and IL-6. In the last few years there has been a growing interest to understand the connection between HIF and antimicrobial extracellular traps (ETs) of mast cells and neutrophils. A series of recent studies has indicated that HIF is important for the formation of DNA traps, and its activation seems to augment the antimicrobial defense in mice and humans [59,60]. Regarding the role of HIFs in eosinophils in regulating asthma induction and pathogenesis, Crotty Alexander et al. [61] have shown, that HIF-1α and HIF-2α are involved in the chemotactic properties of eosinophils. The eosinophil-specific deletion of HIF-1α diminishes their chemotactic proprieties in vitro in contrast to eosinophil-specific deletion of HIF-2α.

The metabolism of mast cells, basophils and eosinophils remains poorly understood. Sumbayev et al. [122] demonstrated that these granulocytes use glycolysis as a main source of ATP generation. They have observed that HIF-1α plays a pivotal role in supporting IgE-mediated inflammatory responses of basophils by regulating the release of the cytokine IL-4 and pro-angiogenic VEGF. They also suggest that HIF-1α maintains basophil activity over long periods of inflammation in mice and humans [28,55,122]. Further research studies on mast cells, basophils and eosinophils are desirable to extend our knowledge on how their function and metabolism are modulated by hypoxia and the HIF signaling pathway.

### 3.2. Hypoxia and Macrophages

Macrophages are key effectors of the innate immune response, which can be divided into M1 (classic) and M2 (regulatory) phenotypes. Classically activated macrophages (M1 phenotype) play an important role in the first line of defense against bacterial infections and they produce large amounts of nitric oxide (NO) by inducible nitric oxide synthase (iNOS) [80]. In contrast, alternatively activated (M2 phenotype) have been shown to play an important role in wound healing, tissue repair and regeneration. They are activated by IL-4 and IL-13 and possess anti-inflammatory and pro-angiogenic functions. M2 macrophages produce arginase-1 (Arg-1), Fizz-1, chitinase-like protein 3 and IL10 [123,124]. Hypoxia is a hallmark of solid tumors and a potent driver of malignancy. Macrophages are attracted to hypoxic regions and intratumoral hypoxia plays an important role in the regulation of tumor-associated macrophages (TAMs). In the last decade TAMs have attracted much attention, as TAMs affect several aspects of tumor progression, such as tumor cell survival and angiogenesis. In addition they are engaged in the interaction with different cells of the adaptive immune system, such as T cells [125].

Macrophage-specific deletion of HIF-1α disturbs the production of ATP, which adversely affects survival, invasion, motility, aggregation and bactericidal activity of murine and human macrophages [45,48,81]. Importantly, mice with myeloid cell-specific deletion of HIF-1α are resistant to LPS-induced lethality [50]. This suggests that HIF-1α plays an important role in M1 polarization [82]. Quite recently, considerable attention has been paid to Cdc42 and Rac1 expression mediated by HIF-1α in human and mouse macrophages [126]. Previous research has shown that HIF-1α enhance macrophage migration to the site of infection by increasing the expression of CXCR4 [127] and decreasing CCR5 [128] expression, which leads to macrophage retention in the area of infection. There is evidence that the activation of HIF-2α affects the expression of Arg-1 and favors the M2 phenotype [88]. However, it is not entirely known whether HIF-2α is actually a required element for M2 polarization. Imtiyaz HZ et al. [89] demonstrated that mice lacking HIF-2α in myeloid cells decrease their pro-inflammatory cytokine response after M1 activation and are resistant to LPS-induced endotoxemia. The results obtained by Casazza et al. in [129] suggest that hypoxia drives the pro-tumoral function of M2-like TAMs. They demonstrated that macrophages are attracted into hypoxic tumors by hypoxia-induced, tumor-derived Semaphorin 3 and through neuropilin 1 signaling on macrophages. Once arrived in the hypoxic areas, macrophages are retained and develop a pro-tumorigenic phenotype that facilities angiogenesis, metastasis and immunosuppression.

Classically activated human and murine macrophages (M1 phenotype) use HIF-1α-controlled glycolysis to produce energy [80]. However, it was demonstrated that under normoxia HIF-1 is stabilized by an unexpected function of the membrane type 1 matrix metalloproteinase (MT1-MMP), which binds the FIH-1 and may affect its activity. Activation of HIF-1 by MT1-MMP enhances the rate of aerobic glycolysis of macrophages and finally leads to the stimulation of ATP production via glycolysis [130]. Whereas alternatively activated (M2 phenotype) macrophages adapt oxidative phosphorylation to fuel their long-term functions. The activation and M1 polarization of macrophages with LPS leads to a perturbed Krebs cycle, and HIF accumulation, which promotes glycolysis in which the enzyme hexokinase 1 (HK1). Subsequently, activation of the NLRP3 inflammosome and caspase 1 induce the processing of pro-IL-1β. This leads to citrate accumulation, and finally to generation of prostaglandins, NO and ROS [49,83,84,85]. Accumulating evidence indicates that the TCA cycle intermediates fumarate and succinate modulate HIF-1α activation and the function of macrophages. This has led to the recognition of the role that metabolites can play outside their classical function. In summary, this shows that metabolites guide immune responses and that HIFs contribute to this metabolic reprogramming in macrophages.

### 3.3. Hypoxia and Dendritic Cells

Dendritic cells (DCs) serve as a bridge between innate and adaptive immunity and the role of HIF isoforms has been well studied in these cells [131,132]. DCs can be divided in plasmacytoid dendritic cells (p-DCs) and classical dendritic cells (c-DCs). In a steady state immature pDCs are characterized by relatively low expression of the type I transmembrane glycoprotein CD11c and MHC-II. They show also a limited variety of expression of innate immune receptors called pattern-recognition receptors (PRRs), such as Toll-like receptors (TLRs) 7 and 9. Upon activation, they are able to produce large amounts of type I IFN and effectively present foreign antigens [133,134,135]. They possess a unique ability to detect tissue injuries and antigens. cDCs are masters in presenting phagocytosed antigens to T cells and they are very important cells that control immunity and enforce tolerance to self-antigens [136,137,138,139,140].

It has been shown that activation of the HIF signaling pathway affects several DC functions, including survival, differentiation, maturation, migration and antigen presentation. Moreover, it was confirmed that HIFs are essential regulators of interferon γ (IFN-γ) synthesis, as well as IL-22 and IL-10 production in human and murine DCs [62,63,64,65]. Additionally, hypoxia upregulates the expression of various neutrophil-attracting chemokines, such as CXCR2, CXCR3, CCR5 and CXCL8 in human and murine DCs [67]. Previous studies [68] have also emphasized a protective role for HIF-1α in a dextran sodium sulfate (DSS)-induced model of murine colitis, which is a mouse model of Inflammatory bowel disease (IBD). Dendritic cell-specific depletion of HIF-1α led to a significantly higher loss of body weight, more severe intestinal inflammation, and increased production of pro-inflammatory cytokines in mice with DSS-induced colitis than in WT mice [68]. This increased susceptibility to colitis was also associated with impaired development of regulatory T cells (Tregs).

Metabolic reprogramming plays an important role during dendritic cell activation. Recently published data reported that DC activation by bacterial lipopolysaccharide (LPS) under normoxia promotes production of HIF-1α to greater levels than the levels induced by hypoxia itself [66]. Furthermore, inhibition of glycolysis by 2-DG (the glucose analogue 2-deoxyglucose) affects the DC maturation process in response to LPS stimulation. The accumulation of HIF-1α is crucial for this process, due to the subsequent induction of glycolytic HIF target genes such as GLUT1 [66]. Many aspects of DC metabolism in hypoxia have yet to be elucidated, but the current data suggests that HIF-mediated metabolic reprogramming is involved in the differentiation, proliferation and apoptosis of DCs.

## 4. The Effect of Hypoxia on Adaptive Cell Function and Metabolism

### 4.1. Hypoxia and T Cells

The main function of T lymphocytes is controlling the adaptive immune response against infection and cancer. T cells are formed from lymphoid progenitors in the bone marrow and then migrate to the cortex of the thymus to complete their maturation process [141]. There, T lymphocytes differentiate into various subpopulations of helper CD4+ T cells and CD8+ cytotoxic T cells. Stimulation of naïve CD4+ T cells with specific antigen and the cytokine signals present in the microenvironment causes cell differentiation into the different subpopulations TH1, TH2, TH17 or Treg (regulatory T cells) [141,142]. TH1 cells play a very important role in the protection against viral, bacterial and parasitic infection, in addition to anti-tumoral functions. They require interferon-γ (IFN-γ) and IL-12 for their differentiation. TH2 cells require IL-4 to drive immunity and protection against extracellular parasites. TH17 cells are induced by IL-6 and TGF-β in a HIF-1α-dependent manner, they play an important role in autoimmune diseases [143,144]. The retinoic acid-related orphan receptor γt (RORγt) is the key transcription factor that drives TH17 differentiation [145]. There is some evidence, that the mTOR-HIF-1α axis controls TH17 development, differentiation through transcriptional activation of RORγt and sustained glycolysis [92]. CD8+ T lymphocytes play an essential role in the control of chronic infections and cancer. They become armed effector cells upon confrontation with target cells, which express cognate antigen. Upon activation, CD8+ T cells release cytotoxic granules containing perforin and granzymes that induce target cell apoptosis, and they produce pro-inflammatory cytokines like TNF-α and IFN-γ.

It has been shown that HIF-1α plays a crucial role in regulating survival in human T cells [146]. This data was further confirmed by Biju et al. [102] by using mouse models with conditional knock-outs of HIF-1α and VHL. The HIF-1α pathway is also important for Treg differentiation and function through binding of the Treg-specific transcription factor Foxp3 and its subsequent degradation [93,94]. It is known that human and murine TH17 cells favor glycolysis, while Treg cells preferentially use oxidative phosphorylation and FAO [93,147,148]. Noteworthy, HIF-1α tips the balance between TH17 and Treg differentiation, and indirectly promotes autoimmunity in patients [149,150,151,152,153,154,155,156,157,158].

HIF is essential for controlling the survival, differentiation, proliferation and antitumor capacity of human and murine CD8+ T cells [99]. HIF-1α activation in CD8+ T cells favors a glycolytic metabolism, which is indispensable for effector function [100] and the generation of a tumoricidal memory CD8+ T cell population [101]. A recently published study of Palazon et al. [159] suggests that hypoxia and the HIF signaling pathway impact on tumor infiltration of human cytotoxic T cells. T cell specific deletion of HIF-1α resulted in reduced tumor infiltration and killing, as well as an altered tumor vascularization. Moreover, a study on the T-cell specific role for HIFs in intestinal inflammation showed that HIF-1α is protective in DSS-induced colitis [104] with increased infiltration of TH1 and TH17 in T-cell specific HIF1α knockout mice [104].

Quiescent naïve human and murine T cells use oxidative phosphorylation as main source of energy in contrast to activated T cells, which switch to aerobic glycolysis and glutamine catabolism for nucleotide, lipid, and amino acid production [91,160,161,162]. Several groups have recently explored a potential role of HIFs in the context of adaptive immunity. They have found that pro-inflammatory TH1, TH2 and TH17 lymphocytes after in vitro stimulation preferentially use glycolytic metabolism, contrary to in vitro activated regulatory T cells, which display increased lipid oxidation and oxidative phosphorylation [93,163]. Very interesting data have recently been published on type I regulatory T cells (Tr1 cells), which are regulated by the HIF signaling pathway. They described that HIF-1α controls the early metabolic reprogramming of human and murine Tr1 cells [97]. They show that aerobic glycolysis promotes Tr1 cell differentiation through a metabolic program regulated by HIF1-α and AHR. Additionally, they report that oxygen and extracellular adenosine triphosphate (eATP) control the differentiation of Tr1 cells through HIF1-α dependent manner. These cells are a Foxp3-neg subpopulation of Tregs with the capacity to attenuate TH17 cells via the release of IL-27 [98]. A recently published study of Phan et al. [164] demonstrated that constitutive glycolytic metabolism in CD8+ T cells induced by conditional knock-out of Vhl promotes accelerated CD8+ memory cell differentiation against viral infection and supports the formation of long-lived effector-memory CD8+ T cells. In summary, the recent interest in the role of HIF signaling in T cells has yielded remarkable advances in our understanding of how hypoxia regulates T cell function.

### 4.2. Hypoxia and B Cells

B cells and their antibodies play a crucial role in humoral immunity and the protection against an almost unlimited variety of pathogens. Imperfections in the B-cell development process lead to autoimmunity, malignancy, immunodeficiency, or allergy [165,166]. In addition to antibody production, B cells release a broad variety of cytokines (IL-35, TGF-β, and in particular IL-10) [167,168,169] and contribute to immunomodulatory responses [166,170].

Hypoxia and the HIF signaling pathway play a very important role in B cell development and function. It has been proven that the absence of HIF-1α in the lymphoid tissues of chimeric mice causes abnormal B cell development and autoimmunity [109], via the impairment of hypoxia-induced cell cycle arrest in B cells [111]. Recently, it was published that HIF-1α plays a crucial role in the expression of TASK-2 potassium channels in B cells, which are essential for many B cell functions, such as proliferation, survival or cytokine production [110].

Hypoxia and the HIF signaling pathway increase glycolytic metabolism in germinal center (GC) B cells, which affects B cell proliferation, apoptosis and antibody production [105]. It was reported that constitutive activation and stabilization of HIF-1α by conditional B-cell-specific deletion of VHL leads to B-cell proliferation, decreases antigen-specific germinal center (GC) B cells and impairs the generation of high-affinity IgG antibodies [105,106]. Jellusova et al. [107] demonstrated that GC B cells, increase glycolysis and mitochondrial biogenesis via HIF and glycogen synthase kinase 3 (Gsk3), respectively, during metabolic adaptation. They have found that Gsk3 was necessary for the generation and maintenance of GC B cells to maintain high glycolytic activity for the growth and proliferation in hypoxic conditions. The role of HIF target genes in murine and human B cell metabolism was confirmed by Caro-Maldonado A et al. [108], who demonstrated that B cell-specific deletion of Glut1 led to reduced B cell proliferation and impaired antibody production in vivo. A recently published study of Meng et al. [171] show*s* a novel molecular mechanism for the regulation of autoimmune disease by CD1d high CD5+ B cells. This elegant study suggests that via the modulation of glycolytic metabolism, HIF-1α has an impact on this specific population of B cells. They demonstrated that the HIF signaling pathway directly impacts the IL-10 production by B cells. In consequence, HIF-1α activation in B cells regulates autoimmune diseases such as experimental autoimmune encephalomyelitis (EAE) and arthritis. In summary, a deeper understanding of the HIF pathway in B cells is desirable and may lead to therapeutic modulation of immune responses during vaccination and autoimmune diseases.

## 5. The Effect of Hypoxia on Innate Lymphoid Cell Function and Metabolism

### 5.1. Hypoxia and ILC1 Cells

Innate lymphoid cells (ILCs) are a recently discovered immune cell type, which plays an important role in lymphoid organogenesis, epithelial tissue homeostasis and defense, as well in the amplification of inflammatory responses [105,170]. Group 1 ILCs includes conventional Natural Killer (NK) cells and non-NK cell ILC1, which are characterized based on their ability to produce INF-γ and TNF-α in response to stimulation with IL-12, IL-15, or IL-18, and expression of the transcription factors T-bet and EOMES [172]. They play an important role in promoting responses against intracellular pathogens such as Toxoplasma gondii [173]. NK cells are a subset of cytotoxic ILC1 with unique anticancer and antiviral activity [174,175,176,177]. NK cells carry out direct cytotoxicity of target cells via the release of Granzymes and Perforins, regulate immune responses via cytokine production (TNFα and INF-γ) and influence DC maturation [178].

Our recent research showed that the tumor infiltrating NK cells operate in hypoxic microenvironments and we have demonstrated that HIF-1α is required for cytokine production and target cell killing upon NK cell activation, whereas the absence of HIF-1α impairs NK cell activation and effector potential. The deletion of HIF-1α in NK cells also lead to increased bioavailability of the major angiogenic cytokine vascular endothelial growth factor (VEGF), which was due to decreased numbers of tumor infiltrating NK cells that express angiostatic soluble version of Vascular Endothelial Gowth Factor Receptor 1 (VEGFR-1). Surprisingly, this resulted in non-productive angiogenesis, the creation of a high-density network of immature vessels, severe tumor hemorrhage and repressed tumor growth [70]. In line with our data, it has been reported that hypoxia suppresses the cytotoxic potential of human NK cells against multiple myeloma, which can be restored by IL-2 activation [72]. Moreover, it has been also shown by Sceneay et al. [75] that hypoxia impairs NK cell cytotoxicity. They discovered that tumor hypoxia caused the reduction in cytotoxic potential of NK cells, resulting in a decreased antitumor response that allowed metastasis formation in secondary organs. In contrast, metastatic burden was reduced when active NK cells were present in pre-metastatic lungs [75]. Current research also shows that hypoxia via tumor-derived microvesicles (TD-MVs) downregulates the expression of MICA (NKG2D ligand) on tumor cells, and the activating receptor NKG2D expression on human and murine NK cells [73,74]. These tumor-derived microvesicles negatively regulate NK cells function by impaired CD107a expression via a miR-23a dependent mechanism. This is the first study to demonstrate that hypoxic tumor cells by secreting MVs can educate NK cells and impair their antitumor immune response [73]. Interestingly, in another study it was shown that hypoxia-induced autophagy reduces breast cancer cell susceptibility to NK cell-mediated lysis. However, this process is reversible after targeting autophagy in tumor cells [77,78]. Finally, hypoxia has an important impact on the antiviral function of NK cells from HCV(+) patients [76].

In analogy to naïve human and murine T cells, resting NK cells predominantly use oxidative phosphorylation over aerobic glycolysis prior to activation [172]. Naïve NK cells possess limited requirements and they metabolize glucose through glycolysis coupled to oxidative phosphorylation to make ATP. This was confirmed by transcriptional analysis in which resting NK cells were enriched for genes associated with oxidative phosphorylation, fatty acid oxidation and autophagy [173,174], and short-term activation (4–6 h) in the presence of cytokines or activating ligands did not significantly alter the metabolic pathways used by NK cells. However, the metabolic profiling after extended stimulation with high dose IL-15 (100 ng/mL for 3–5 days) of in vitro activated NK cells shows induction of both glycolysis and oxidative phosphorylation. The priming with IL-15 was essential for significant induction of glycolysis [173,174]. In addition, Velasquez et al. [175] recently reported that NK cell activation under hypoxia compared with normoxia in the presence of IL-15 priming synergistically increased glycolytic gene expression without major changes in glycolytic flux and glucose consumption. Upon long-term activation, murine and human NK cells rely on glucose metabolism via aerobic glycolysis in which they metabolize glucose into lactate [173,176,177,178]. IL2, produced by activated T cells, is the most important cytokine in activation of NK cells, which drives glycolytic reprogramming of both murine and human NK cells dependent on the mammalian target of rapamycin complex 1 (mTORC1) [172,173,176,178]. mTORC1 is a key regulator of immunological and metabolic responses, but the exact mechanism how mTORC1 regulates and controls NK cell metabolism is currently poorly understood. Presumably, HIF-1α and cMyc cooperate during this process [28,69]. Finally, a bimodal model for NK cell immune function and metabolic reprogramming was proposed [69]. The first part of the model represents circulating human and murine NK cells, which respond rapidly to activation signals from early innate cytokines (IL12, IL15, and IL18) and do not cause major changes in cellular metabolism. However, the second part of the proposed model represents NK cells that are activated for extended time periods. IL2, which is mainly produced by activated T cells, is crucial cytokine in this process, which drives mTORC1-dependent glycolytic reprogramming of NK cells and creates a bridge between the adaptive and innate immune response.

### 5.2. Hypoxia ILC2 and ILC3.

Innate lymphoid cells type 2 (ILC2) express the transcription factor GATA-3, can respond to IL-25 and IL-33, and produce the type 2 cytokines IL-5 and IL-13 [179,180]. Several studies have shown that ILC2 contribute to immunity against helminth infections, tissue repair, allergic inflammation and asthma [181,182,183,184]. Innate lymphoid cells type 3 (ILC3) are divided in two subpopulation, the CCR6+ lymphoid-tissue inducer (LTi) ILC3 cells and the CCR6- ILC3 cells (NKp44+ in human [185] and NKp46+ in mice [186]). They are characterized by the expression of the nuclear hormone retinoic acid receptor-related orphan receptor γt (RORγt) and production of IL-22 and IL-17 in response to IL-23 or IL-1β [187]. ILC3 play a crucial role in mucosal immune defense [184].

In analogy to naïve ILC1 cells, resting ILC2 and ILC3 cells predominantly adopt oxidative phosphorylation over aerobic glycolysis prior to activation [172]. However, following IL-33 stimulation, activated ILC2 cells augment aerobic glycolysis, and use arginase-1 (Arg-1) to metabolize extracellular L-arginine. Yet, the deletion of Arg-1 affected neither lung ILC2 proliferation nor expression of cytokines IL-5 and IL-13 after helminth infection. Arg1 gene expression can be induced by hypoxia [188], however this raises the question of how Arg1 activity is regulated under hypoxia in ILC2, and how HIFs participate in this regulation. A recently published study by Li et al. [79] suggests that hypoxia and the HIF signaling pathway directly impacts on the late stage of maturation and function of ILC2 cells via the IL33-ST2 pathway. This very elegant study suggests that the VHL-HIF-1α pathway plays a very important role as ca heckpoint for the terminal differentiation of ILC2 located in peripheral organs such as the intestines, lungs or adipose tissue. Further research on ILC2 and ILC3 is desirable to extend our knowledge of how their function and metabolism are modulated by hypoxia and the HIF signaling pathway.

## 6. Conclusions

It can be concluded that the HIF signaling pathway plays an important role in the function of virtually all immune cells via metabolic reprogramming (as shown in Table 1 and Figure 2). Hence, the crosstalk between the metabolic regulation, hypoxia and immunological responses represents a novel and promising therapeutic target.

## Figures and Tables

**Table 1:** The role of hypoxia-inducible factors in migratory immune cells.

Cell Type	HIFα-Mediated Effects
**basophils, eosinophils, mast cells**	**HIF1:** Survival and function, chemotaxis, IL-8 and TNF-α production, stimulation of VEGF, CXCL8 and IL-6 production, formation of DNA traps [55,56,57,58,59,60,61] **HIF2:** Chemotaxis [61]
**dendritic cells**	**HIF1:** Survival, migration, pro-inflammatory cytokine (INF, IL-22, IL-10) production, differentiation, activation, T cell stimulation, antigen presentation [62,63,64,65,66,67,68]
**innate lymphoid cells** **ILC1 and NK cells, ILC2, ILC3**	**HIF1 (NK cells):** Metabolic reprogramming, impact on tumorigenic and viral potential, impact on number of tumor infiltrating NK cells expressing sVEGFR1, impact on cytotoxic function [28,69,70,71,72,73,74,75,76,77,78] **HIF1 (ILC2):** impact on the late stage of maturation and function via IL33-ST2 pathway [79]
**macrophages** **Classic M1 macrophages, Regulatory M2 macrophages**	**HIF1:** M1 polarization, motility, aggregation, invasion, metabolism, phagocytosis, chemotaxis, bactericidal activity, tumorigenic potential, expression of pro-inflammatory cytokines, increased TLR4 expression [45,48,49,50,80,81,82,83,84,85,86,87] **HIF2:** M1 polarization, motility, metabolism, bactericidal activity, tumorigenic potential [88,89]
**neutrophils**	**HIF1:** Survival, migration, invasion, bactericidal activity, promoted the on-state, increased pro-inflammatory cytokine production and nitric oxide [45,46,47,48,49,50,90] **HIF2:** Survival, increased resistance to nitrosative stress (Catalase) [46,54]
**T cells** **T helper CD4+,** **Cytotoxic CD8+ T cells**	**HIF1:** TH17 and Treg differentiation, survival, proliferation, migration, metabolic reprogramming; CD4+: increased IL17A production; CD8+: increased cytolitic activity, granzyme and perforin production and expression of costimulatory/inhibitory molecules (CTLA-4, GITR, 4-1BB) [91,92,93,94,95,96,97,98,99,100,101,102,103,104] **HIF2:** T cell suppression (Arginase), impact on thymocyte development [46]
**B cells**	**HIF1:** Abnormal B-cell development, impact on proliferation and cell death, autoimmunity, ion transfer, enhanced IgG2c production [105,106,107,108,109,110,111] **HIF2:** impact on proliferation and cell death, enhanced IgG2c production [105,109,111]
